# Plasma orexin-A levels in women with postpartum depression: a prospective controlled study

**DOI:** 10.1590/1806-9282.20252018

**Published:** 2026-06-15

**Authors:** Mustafa Kaplanoglu, Gulcin Daglioglu

**Affiliations:** 1 Medical Park Adana Hospital, Departments of Obstetrics and Gynecology – Adana, Turkiye.; 2 Cukurova University, Faculty of Medicine, Department of Biochemistry – Adana, Turkiye.

**Keywords:** Postpartum depression, Orexin, Hypocretin, Biomarker, Mental health

## Abstract

**OBJECTIVE:**

Postpartum depression is a common mood disorder. Orexin-A, a hypothalamic neuropeptide involved in regulating arousal, stress response, and emotional processes, is implicated in major depressive disorder. The aim of this study was to assess plasma orexin-A levels in women with postpartum depression and to explore its potential biological involvement in postpartum depression.

**METHODS:**

A prospective controlled pilot trial was conducted at two hospitals between April 1, 2024, and June 30, 2025. In total, 60 postpartum women were included: 30 women with postpartum depression, defined as Edinburgh Postpartum Depression Scale ≥13 and confirmed by psychiatric evaluation, and 30 age- and parity-matched healthy controls (Edinburgh Postpartum Depression Scale <13, no psychiatric diagnosis). Venous blood samples were collected 1 month after delivery, and plasma orexin-A was measured. Demographic, obstetric, and clinical characteristics were recorded.

**RESULTS:**

The groups were comparable in terms of demographic and obstetric characteristics. Mean plasma orexin-A levels were significantly lower in women with postpartum depression compared to controls (65.2±10.8 vs. 79.9±6.4 pg/mL, p=0.021). Orexin-A levels were positively correlated with maternal age (r=0.328, p=0.011) but were not associated with parity, body mass index, neonatal parameters, or family history.

**CONCLUSION:**

This preliminary clinical evidence demonstrates that plasma orexin-A levels are reduced in women with postpartum depression, independent of major demographic or obstetric factors. The findings support orexin-A as a potential biomarker and therapeutic target in postpartum depression. Larger longitudinal studies are needed to confirm causality and investigate orexin-based interventions.

## INTRODUCTION

Depression is one of the most common mental health problems worldwide, affecting approximately 12–20% of women and occurring approximately twice as frequently in women as in men^1,2^. Higher rates in women occur during pregnancy and the postpartum period, particularly when hormone levels are rapidly changing^3,4^. These biological and emotional transitions are crucial for the health of both mother and baby.

Postpartum depression (PPD), which falls within the spectrum of major depressive disorder (MDD), is characterized by persistent sadness, loss of interest, fatigue, and sleep and appetite disturbances that appear within the first year after birth. Diagnosis is made according to DSM-5 criteria, and the Edinburgh Postnatal Depression Scale (EPDS) is often used for screening. Depending on cultural and social factors, approximately 10–15% of mothers experience PPD^5^.

In addition to its impact on maternal mental health, PPD can also affect the emotional bond between mother and baby and harm child development. It has been associated with long-term emotional and cognitive difficulties in children, and postpartum suicide accounts for approximately 20% of maternal deaths^5,6^. Neuroimaging studies have also shown that infants of mothers with PPD may have delayed hippocampal growth, reduced cortical thickness, and altered prefrontal–amygdala connections^7^.

The development of PPD is complex and is influenced by a variety of factors, including hormonal changes, genetics, stress, and neurotransmitter imbalances. The sudden drop in steroid hormones after birth is thought to disrupt the neuroendocrine system and trigger depressive symptoms^8^.

Orexin (OX), a neuropeptide produced in the hypothalamus and discovered in 1998, plays a key role in many bodily processes. Sakurai and colleagues named it "orexin" for its role in stimulating appetite, while De Lecea and colleagues used the name "hypocretin" to emphasize its hypothalamic origin. OX-A and OX-B act through OX1R and OX2R receptors to help regulate sleep, energy balance, and emotional control^9^. Low levels of OX-A have been observed in the cerebrospinal fluid (CSF) of individuals with MDD, and experimental blockade of orexin receptors has been shown to induce depression-like behaviors^8^. These findings suggest that inadequate adaptation of the orexin system after birth may contribute to PPD; however, no clinical studies have directly measured plasma OX-A levels in affected women.

This study aimed to measure plasma OX-A levels in women diagnosed with PPD and investigate its possible role in the mechanisms underlying the disorder. Because orexins are known to play a role in mood regulation and stress adaptation, maintaining normal orexin function may be important for emotional stability in the postpartum period. The current lack of clinical data on OX levels in postpartum depression highlights the novelty of this study and its potential contribution to identifying biomarkers that could support early diagnosis and lead to the development of orexin-based treatment approaches.

## METHODS

### Study design and participants

This prospective, controlled pilot study was conducted between April 1, 2024, and June 30, 2025, at the Departments of Obstetrics and Gynecology of Adana Medicalpark Hospital and Yüreğir State Hospital. The study was designed to evaluate the feasibility and generate preliminary data regarding the association between serum OX-A and postpartum depression. A total of 60 postpartum women were included in the study: 30 women diagnosed with PPD and 30 healthy age- and parity-matched controls. Written informed consent was obtained from all participants. The study was conducted in accordance with the principles of the Declaration of Helsinki and approved by the Ethics Institutional Review Board of Çukurova University Faculty of Medicine (Approval No: 131/2023).

As part of routine clinical care, all postpartum women underwent screening with the EPDS prior to hospital discharge. Women who subsequently attended postpartum follow-up visits were assessed for eligibility for the present study. Participants were invited to follow-up visits at 1 week and 1 month postpartum. EPDS scores obtained during routine screening were used to classify participants. Women with an EPDS score ≥13 and a confirmed diagnosis of postpartum depression by a psychiatrist were included in the PPD group. Women with an EPDS score <13 who met the study criteria were included in the control group.

### Inclusion and exclusion criteria

Eligible participants were women with a term singleton delivery who had completed at least primary school, provided written informed consent, and attended a 1-month postpartum follow-up visit.

Participants were categorized according to their EPDS scores: those with a score of ≥13 constituted the PPD group, and those with a score of <13 constituted the control group.

Exclusion criteria included a history of psychiatric disorders or use of psychotropic medications (antidepressants or antipsychotics), acute or chronic systemic disease (e.g., significant cardiac, hepatic, renal), endocrine disorders (e.g., uncontrolled diabetes, thyroid dysfunction), pregnancy complications (preeclampsia, gestational diabetes, premature rupture of membranes, postpartum hemorrhage), preterm birth, congenital anomalies, multiple gestation, or need for neonatal intensive care.

### Blood sample collection and processing

Venous blood samples (6 mL) were collected between 11:00 a.m. and 12:00 p.m. from the antecubital vein into EDTA tubes and centrifuged at 2,000 rpm for 20 min (Nüve NF 1200). Plasma was aliquoted into sterile Eppendorf tubes and stored at −80°C until analysis. Before measurement, samples were thawed at +4°C and homogenized using a Vortex-Genie 2. Plasma orexin-A (OX-A) levels were determined by competitive ELISA (Elabscience^®^, Houston, TX, USA; Cat. No. E-EL-H1015) on a semi-automated microplate reader (CHROMATE, Awareness Technology, FL, USA). Serum hormone profiles were analyzed by chemiluminescent microparticle immunoassay (CMIA) on the Abbott Architect i1000SR. The ELISA assay had a sensitivity of 37.5 pg/mL, a measurement range of 62.5–4,000 pg/mL, and intra/inter-assay CVs<10%. Mean recovery was 101% in EDTA plasma, 97% in serum, and 95% in supernatants.

### Clinical assessment and follow-up

Clinical assessments were performed by researchers at the designated outpatient clinics. All parameters (pregnancy history and laboratory assessments), except for OX-A measurement, were assessed as part of routine postpartum care. No participants received psychotropic medications during the study period.

### Sample size and statistical analysis

A total of 60 postpartum women (30 with PPD and 30 controls) were included in the study. The sample size was determined based on methodologies derived from previous studies examining the association between PPD and biomarkers. This study was designed as an exploratory study, and the findings are intended to guide future, larger-scale studies.

### Statistical analysis

Statistical analyses were performed using IBM SPSS Statistics for Windows, Version 26.0 (IBM Corp., Armonk, NY, USA). The distribution of continuous variables was assessed using the Shapiro-Wilk test. Normally distributed data are presented as mean±standard deviation, while non-normally distributed data are expressed as median (minimum–maximum). Differences between the two groups were analyzed using the independent samples t-test or Mann-Whitney U test, as appropriate. Categorical variables were expressed as counts and percentages and compared using the chi-square or Fisher's exact test. Logistic regression analysis was performed to examine the association between OX-A levels and PPD. A p<0.05 was considered statistically significant.

## RESULTS

A total of 60 postpartum women were included in the study: 30 with PPD and 30 healthy controls. Baseline demographic and obstetric characteristics were largely similar between the groups (Table 1). No significant differences were observed in maternal age, gestational age at delivery, parity, number of living children, family history of PPD, total gestational weight gain, or neonatal sex. Hemoglobin and hematocrit levels were also similar. Although body mass index (BMI) was higher in the PPD group than in the control group, this difference was not statistically significant (25.9±3.0 kg/m^2^ vs. 24.8±2.4 kg/m^2^, p=0.058). The distribution of educational level was also similar (p=0.6). In contrast, plasma OX-A levels were significantly different between the groups. Plasma OX-A concentrations in women with PPD were significantly lower compared to healthy controls (65.2±10.8 pg/mL vs. 79.9±6.4 pg/mL; p=0.021).

**Table 1:** Demographic and clinical characteristics of women with postpartum depression and controls.

Variable		PPD (n=30)	Control (n=30)	p-value
Maternal age (years) (mean±SD)		27.4±4.7	29.6±4.1	0.166
Gestational age (weeks) (mean±SD)		38.1±1.5	37.9±1.4	0.194
Gravidity, median (IQR)		2 (1–3)	2 (0–2)	0.772
Parity, median (IQR)		1 (0–1)	1 (0–1)	0.179
Number of live births, median (IQR)		1 (0–1)	0 (0–1)	0.150
Family history, n (%)		25 (83.3)	23 (76.6)	0.748
BMI (kg/m^2^)		25.9±3.0	24.8±2.4	0.058
Educational level, n (%)				
	Primary	7 (23.3)	12 (40.0)	0.600
	Middle	6 (20.0)	6 (20.0)	
	High	9 (30.0)	4 (13.3)	
	Undergraduate	4 (13.3)	5 (16.7)	
	Postgraduate	4 (13.3)	3 (10.0)	
Neonatal gender, n (%)				
	Male	16 (53.3)	15 (50.0)	0.796
	Female	14 (46.7)	15 (50.0)	
Hemoglobin (g/dL) (mean±SD)		10.9±1.2	11.0±1.3	0.628
Hematocrit (%) (mean±SD)		31.8±2.7	31.1±2.2	0.094
Orexin (pg/mL) (mean±SD)		65.2±10.8	79.9±6.4	0.021
Gestational weight gain (kg) (mean±SD)		10.2±5.7	9.5±5.2	0.767

PPD: postpartum depression; MA: menarche age; BMI: body mass index; Hgb: hemoglobin; Hct: hematocrit; OX: orexin; SD: standard deviation; IQR: interquartile range.

Correlation analysis revealed a significant and positive association only between maternal age and OX-A levels (r=0.328, p=0.011) (Table 2). No significant correlation was observed between OX-A levels and gestational weight gain, family history of PPD, mode of delivery, newborn birth weight, maternal BMI, or newborn sex.

**Table 2:** Comparison of correlation analysis between groups.

Parameter	r	p-value
Maternal age	0.328	0.011
Gestational weight gain	-0.237	0.652
Family history	0.086	0.513
Mode of delivery	0.134	0.307
Fetal Birth weight	0.030	0.981
Maternal BMI	0.003	0.982
Neonatal gender	0.064	0.629

BMI: body mass index; MA: menarche age.

These findings indicate that plasma OX-A levels are significantly reduced in women with PPD and that this reduction is independent of major demographic or obstetric factors. The positive correlation with maternal age suggests a potential moderating effect of age on OX regulation in the postpartum period.

## DISCUSSION

In this study, we found that women with PPD had significantly lower plasma OX-A levels compared to healthy controls. The lack of significant differences in demographic or obstetric characteristics between the two groups suggests that low OX-A levels may serve as a biomarker independent of these factors. Furthermore, the positive association between maternal age and OX-A levels suggests that the OX system may change with age in the postpartum period. Overall, this study demonstrates an association between postpartum depression and alterations in plasma orexin-A levels, suggesting a potential involvement of the orexin system in postpartum mood regulation. As a pilot study, these findings should be interpreted as preliminary and hypothesis-generating rather than confirmatory.

Peripartum depression encompasses depressive symptoms occurring during pregnancy and the postpartum period, whereas postpartum depression refers specifically to depressive episodes emerging after childbirth. Although these conditions share overlapping clinical features, the postpartum period represents a unique biological and psychosocial state characterized by abrupt hormonal withdrawal, neuroendocrine adaptation, and increased caregiving-related stress. In the present study, we deliberately focused on postpartum depression to reduce biological heterogeneity and to better capture neuropeptide alterations specifically related to the postpartum transition. Given the dynamic regulation of the orexin system in response to hormonal and stress-related changes, restricting the analysis to the postpartum period may allow more precise interpretation of the observed associations.

While many different molecules have been investigated in depression, OX-A receptors (OX-A) have come to the fore in recent years^10^. Studies in MDD and anxiety disorders indicate that low OX-A levels are generally associated with more severe symptoms. For example, one study found reduced OX expression in the blood cells of MDD patients and a negative correlation between OX-A mRNA levels and depression scores^11^. Another study reported significantly lower CSF OX-A levels in MDD patients at risk for suicide^12^. At the epigenetic level, hypermethylation of OX gene promoters can reduce peptide production and lead to depressive symptoms^13^. However, there are also studies that find the opposite^14^. Overall, our findings support a negative association between plasma OX-A levels and depressive symptoms in postpartum depression.

Experimental animal studies also show that blocking or suppressing OX receptors causes depression-like behaviors, while OX activation produces anxiolytic and antidepressant effects^8,15^. Given the unique biological stressors of the postpartum period, the low OX-A levels observed in our study population may suggest that an inadequate neuropeptide response contributes to depressive symptoms.

The postpartum period represents a physiological transition characterized by abrupt declines in estrogen and progesterone, increased HPA axis activity, and significant psychosocial stress. OX neurons interact with multiple neurotransmitter systems [serotonin, dopamine, gamma-aminobutyric acid (GABA)] and serve as critical modulators of stress responses and emotional regulation. Ji et al. elevated OX levels under experimental stress conditions have been shown to prevent depressive responses and enhance stress resilience^8^. In contrast, our findings of low OX-A levels in women with PPD suggest an inadequate adaptive response to postpartum stress, potentially facilitating the emergence of depressive symptoms.

The positive correlation between maternal age and OX-A levels is noteworthy. Epidemiological evidence suggests that younger women are at higher risk for PPD; this may be related to decreased OX functionality or decreased sensitivity to hormonal changes during early reproductive age^16,17^. This observation suggests that PPD is associated with some age-related biological mechanism and warrants further investigation. From a biological perspective, maternal age may influence the orexin system through several interrelated mechanisms. Neuroendocrine maturation, cumulative lifetime exposure to ovarian steroid fluctuations, and age-dependent stress adaptation capacity may affect both orexin synthesis and receptor signaling. Younger women may exhibit a less robust orexinergic response to the profound hormonal withdrawal and psychosocial stress that characterize the postpartum period. In addition, age-related differences in hypothalamic plasticity and orexin neuron responsiveness may modulate the ability of the orexin system to buffer stress and regulate mood. Experimental data suggest that orexin interacts closely with the hypothalamic–pituitary–adrenal axis, serotonergic pathways, and dopaminergic reward circuits; reduced efficiency of these interactions at earlier reproductive ages could contribute to lower circulating OX-A levels and increased susceptibility to postpartum depressive symptoms.

To our knowledge, no previous clinical study has specifically examined plasma OX-A levels in PPD. Current screening methods rely primarily on self-report questionnaires (e.g., EPDS) and are subject to limitations such as cultural factors and lack of awareness. Adding biomarkers such as OX-A to the diagnostic process may enable earlier and more objective identification of at-risk individuals. Additionally, the OX system offers a pharmacologically targetable pathway. Dual OX receptor antagonists are in clinical use for sleep disorders, and selective OX agonists are in development for the treatment of specific conditions. These agents may be promising for the treatment of PPD.

Our study has some limitations. As a prospective controlled pilot study with a relatively small sample size, the findings should be interpreted as preliminary and hypothesis-generating. Even if peripheral levels are clinically relevant, plasma measurements may not fully reflect central OX activity. Additionally, simultaneous assessment of hormonal or inflammatory markers was not performed, precluding evaluation of possible interactions with OX.

Despite these limitations, our findings provide the first clinical evidence demonstrating the role of the OX system in PPD. It offers a potential avenue for understanding the biological basis of PPD and new treatment approaches.

## CONCLUSION AND CLINICAL RESULTS

This study demonstrated significantly decreased plasma OX-A levels in women with PPD, providing the first clinical evidence that the orexin system may play a role in the pathophysiology of the disease. The association between OX-A levels and maternal age suggests that age-related neuropeptidergic changes may influence the development of PPD. Incorporating objective biomarkers such as plasma OX-A into clinical assessment may enable early and accurate identification of at-risk individuals. In conclusion, our findings highlight OX-A as a potential biomarker and therapeutic target for PPD and warrant further validation in larger prospective studies.

## Data Availability

The datasets generated and/or analyzed during the current study are available from the corresponding author upon reasonable request.

## References

[1] Greenberg PE, Kessler RC, Birnbaum HG, Leong SA, Lowe SW, Berglund PA (2003). The economic burden of depression in the United States: how did it change between 1990 and 2000?. J Clin Psychiatry.

[2] Li XL, Lin GY, Li KQ, Zhu LJ, Xu LW, Li SN (2025). A meta-analysis on the incidence rate of depression in Chinese menopausal women. BMC Psychiatry.

[3] Ciolac L, Bernad ES, Tudor A, Nițu DR, Buleu F, Popa DI (2025). Postpartum depression: interacting biological pathways and the promising validation of blood-based biomarkers. J Clin Med.

[4] Wu X, Jin R (2025;). Effects of postpartum hormonal changes on the immune system and their role in recovery. Acta Biochim Pol.

[5] Yetwale A, Mulugeta C, Biyazin T, Fenta B, Alamrew A, Emagneneh T (2025). Suicidal ideation and its associated factors among pregnant and post-partum women in Ethiopia, a systematic review and meta-analysis, 2025. BMC Psychiatry.

[6] Payne JL, Maguire J (2019). Pathophysiological mechanisms implicated in postpartum depression. Front Neuroendocrinol.

[7] Duan C, Hare MM, Staring M, Deligiannidis KM (2019). Examining the relationship between perinatal depression and neurodevelopment in infants and children through structural and functional neuroimaging research. Int Rev Psychiatry.

[8] Ji MJ, Zhang XY, Chen Z, Wang JJ, Zhu JN (2019). Orexin prevents depressive-like behavior by promoting stress resilience. Mol Psychiatry.

[9] Sakurai T, Amemiya A, Ishii M, Matsuzaki I, Chemelli RM, Tanaka H (1998). Orexins and orexin receptors: a family of hypothalamic neuropeptides and G protein-coupled receptors that regulate feeding behavior. Cell.

[10] Thase ME (2025). A new direction for adjunctive therapy of difficult-to-treat depression: examining the role of orexin receptor antagonists. Eur Arch Psychiatry Clin Neurosci.

[11] Rotter A, Asemann R, Decker A, Kornhuber J, Biermann T (2011). Orexin expression and promoter-methylation in peripheral blood of patients suffering from major depressive disorder. J Affect Disord.

[12] Brundin L, Björkqvist M, Petersén A, Träskman-Bendz L (2007). Reduced orexin levels in the cerebrospinal fluid of suicidal patients with major depressive disorder. Eur Neuropsychopharmacol.

[13] Pu C, Tian S, He S, Chen W, He Y, Ren H (2020). Depression and stress levels increase risk of liver cancer through epigenetic downregulation of hypocretin. Genes Dis.

[14] Li H, Lu J, Li S, Huang B, Shi G, Mou T (2021;). Increased hypocretin (orexin) plasma level in depression, bipolar disorder patients. Front Psychiatry.

[15] Gotter AL, Roecker AJ, Hargreaves R, Coleman PJ, Winrow CJ, Renger JJ (2012). Orexin receptors as therapeutic drug targets. Prog Brain Res.

[16] Jeon N, Kent-Marvick J, Sanders JN, Hanson H, Simonsen SE (2024). Comparing maternal factors associated with postpartum depression between primiparous adolescents and adults: a large retrospective cohort study. Birth.

[17] Tenaw LA, Ngai FW, Bessie C (2024). Effectiveness of psychosocial interventions in preventing postpartum depression among teenage mothers-systematic review and meta-analysis of randomized controlled trials. Prev Sci.

